# Clinical Reports

**Published:** 1880-05

**Authors:** 


### EDITORIAL.

### CLINICAL REPORTS.

THE most important feature introduced by the present management into the JOURNAL is that of Clinical Reports. Two objects are designed to be subserved in this department: 1st, to encourage in the profession a habit, now sadly neglected, of keeping a record of cases, occuring in daily practice; 2d, to publish and preserve from oblivion, the observations, experiences and practice of physicians in rare and isolated cases, thus forming a mass of carefully collated facts and statistics.

In this department especially, we fulfill the earnest purpose set forth in our prospectus, to make the JOURNAL the reflex of medical thought as well as the exponent of the professional skill and attainments of our readers. With a view to secure so desirable an end it is positively essential that the profession, both hereand elsewhere, should lend their aid in sending communications of their original researches with their practical observations and careful study of cases, ordinary as well as anomalous.

Medical Journals, not devoted to a specialty, but intended for the general practitioner, are valuable, not by reason of long original papers, which may be very hastily and superficially perused, but from practical hints and facts deduced from cases, which have been closely studied and carefully digested, as well as from judicious selections, covering a wide range of medical literature.

Reports, that are instructive and interesting, need not, of necessity, contain anything startling or wonderful. Often the most simple cases, if skillfully treated, convey a hint or an idea to the careful and interested student, of greater practical utility than the rarest instances of disease or the most heroic operations in surgery.

Reports also of comparatively rare diseases and operations, are at least valuable to the statistician, while cases, illustrating some new plan of treatment, or some new method of surgical procedure, are of interest to the thoughtful mind searching for light to clear up obscure cases often met with in professional life.

In this connection we have only one admonition to offer, that our contributors strive to guard against prolixity. We join in the sentiment adopted by secular Press: "Be brief; be brief; ever more be brief." Conciseness is not incompatible with clearness of description. It is not necessary for a thorough comprehension of a case to consume space in a detailed account of the hourly changes of temperature and variations of pulse. In such minuteness of detail, the significance of cases in a surgical, pathological and therapeutical sense may be obscured and their real value destroyed.

Recurring to the objects intended to be subserved in clinical reports, we are conscious that we have in view the real interests of the contributor as well as the greater interests of medical and surgical science.

